# Effectiveness of Village Health Volunteers/Workers Working as Male-Female Pairs on Women’s Use of Postnatal Care Services in Sepone District in Lao People’s Democratic Republic: A Protocol for a Quasi-experimental Cluster Study

**DOI:** 10.7759/cureus.82786

**Published:** 2025-04-22

**Authors:** Noudéhouénou Credo Adelphe Ahissou, Manami Uehara, Daisuke Nonaka, Inthanomchanh Vongphoumy, Tiengkham Pongvongsa, Khamtoun Ounlienvongsack, Khampheng Phongluxa, Sengchanh Kounnavong, Jun Kobayashi

**Affiliations:** 1 Department of Global Health, Graduate School of Health Sciences, University of the Ryukyus, Okinawa, JPN; 2 Department of Maternal and Child Care, Savannakhet Provincial Health Office, Savannakhet, LAO; 3 Department of Primary Health Care, Savannakhet Provincial Health Office, Savannakhet, LAO; 4 Department of Primary Health Care, Sepone District Health Office, Savannakhet, LAO; 5 Department of Health Research, Lao Tropical and Public Health Institute, Vientiane, LAO

**Keywords:** lao pdr, male and female, pairs, postnatal care, village health volunteers/workers

## Abstract

Background: Despite substantial improvements in antenatal care and skilled birth attendance coverage, postpartum service uptake lags in the Lao People's Democratic Republic (Lao PDR). The 2019 Lao Primary Health Care Policy emphasizes the importance of village health volunteers/workers (VHVs/VHWs) working as groups or pairs to promote primary care services, including obstetric care. However, little is known about whether or how the pairing of male and female VHVs/VHWs may encourage better uptake of postpartum care in communities with solid gender norms.

Objective: Therefore, the study aims to assess the effectiveness of male-female VHVs/VHWs working in pairs on women's uptake of facility-based follow-up PNC visits in rural Sepone, Lao PDR.

Methods: A quasi-experimental cluster study will be conducted between July 2024 and October 2026 in 37 selected sites from two districts in the southern Lao PDR. In 19 selected intervention villages in the Sepone district, trained female-male VHVs/VHWs pairs will promote postpartum services, whereas in 18 similar control villages in the Vilabuly district, VHVs/VHWs will implement standard activities for obstetric care promotion. The study obtained ethical approvals from the University of the Ryukyus and the Lao Tropical and Public Health Institute.

Data analysis: Difference-in-difference analyses will be conducted to estimate service coverage pre-post intervention changes in study sites. Then, using mixed-effect binary logistic regressions, we will assess the association between pairing VHVW/s and facility-based follow-up PNC use, which will be adjusted for other covariates and time effects. Finally, we will analyze the context of pairing VHVs/VHWs implementation using coded transcripts of interviews and focus group discussions.

Results: At the end of the study period, we anticipate at least a 10% increase in follow-up PNC visits at intervention sites compared to control sites. The improvement is expected to result from improved mothers' trust and satisfaction with VHVs/VHWs, and enhanced husband support.

Conclusion: The findings of this study will guide the Lao government's strategy to involve both male and female VHVs/VHWs in primary healthcare according to the 2019 Primary Health Care Policy.

## Introduction

Access to equitable, timely, and people-centered quality maternal and child health (MCH) services continues to be a significant global challenge, especially in developing countries [1]. In 2020, approximately 287,000 mothers and five million children under five died worldwide from preventable causes, primarily in sub-Saharan Africa and southern Asia [2,3].

The Lao People's Democratic Republic (Lao PDR) has made substantial progress in recent decades in improving basic obstetric care coverage and reducing maternal and neonatal deaths [4,5]. According to Lao Social Indicator Surveys, the proportion of pregnant women who utilized antenatal care (ANC) provided by a skilled health professional four times increased from 54.2% in 2012 to 89.8% in 2023, and skilled birth attendance coverage rose from 41.5% in 2012 to 79.8% during the same period [6,7]. However, postnatal care (PNC) continues to lag behind other services, with an increase of only 39.5% to 64.2% in the same period. Moreover, almost all PNC occurs immediately after delivery, and in 2017, fewer than 5% of women had any additional PNC visits [8,9]. The postpartum period, which is at least the first 28 days of life, is a crucial period during which effective follow-up care can significantly increase the chance of survival of newborns [10]. The World Health Organization (WHO) recommends at least four appropriately timed PNC sessions for all mothers and newborns, regardless of their birthplace [11].

In resource-limited settings, community health workers (CHWs) constitute a paid or volunteer public health workforce with an essential role in promoting health services and serve as a contact point between communities and health professionals [12]. In Lao PDR, CHWs are categorized as village health workers (VHWs) and village health volunteers (VHVs) based on their level and length of training, as well as their responsibilities in health promotion activities [13,14]. The 2019 Lao Primary Health Care Policy (PHCP) defines a VHW as an individual “who leads and implements disease prevention and health promotion, provides medical care and refers patients; collects the people’s health information as well as reports to a health center monthly” [15]. VHVs support VHWs, participate in health efforts with health facility staff, collect basic epidemiologic information, and promote proper maternal health practices.

Despite the important role CHWs play in promoting reproductive, maternal, and child health services, their acceptance is often challenged by local gender norms, especially when male or female CHWs approach members of the opposite gender for health promotion [16-18]. For instance, in Tanzania, male CHWs were more effective in discussing sexual and reproductive issues with men and engaging with senior family members, while mothers were more likely to disclose their pregnancies early with female CHWs [19]. Several studies have suggested that pairing male and female CHWs can be an effective strategy to navigate gender norm-related nonacceptance of MCH services provided by CHWs [19-22].

The new Lao PHCP also prioritizes strategies that involve male-female VHVs/VHWs working in pairs or groups to increase the quality of health promotion activities in communities and data reporting [15]. However, the only cross-sectional study that reported the association between pairing female-male VHVs/VHWs and improved mental health among postpartum women in Lao PDR could not determine the potential causal effect or its process [20]. In this context, we evaluate the effectiveness and process of pairing female-male VHVs/VHWs to promote the uptake of PNC in rural Lao PDR, which may guide the PHCP.

## Materials and methods

Design

A quasi-experimental cluster study with intervention and control arms is being conducted between July 2024 and July 2026 [23,24]. The evaluation will be pre-post intervention assessments following the type 1 implementation-effectiveness hybrid design [23]. The quantitative component of the study aims to estimate changes in effectiveness indicators, and the qualitative component will explore the context of implementation and community acceptance.

Settings

This study is being conducted in the Sepone and Vilabuly districts in southern Lao PDR as part of the evaluation of the Japanese International Cooperation Agency (JICA) Grassroot Project entitled "Project for Improvement of MCH by Enhancing Women's Empowerment in Poor and Remote Areas in Sepone District." The two districts are located in Savannakhet Province, approximately 600 kilometers from Vientiane, the capital city of Lao PDR, and close to the Vietnam border [25]. The map of study sites in Lao PDR is shown in Appendix A.

Sepone and Vilabuly share similarities, as both are mountainous areas with ethnic groups, including Lao-Tai, Phutai, Tri, and Makong, and poverty rates of 44.0% and 40.8%, respectively, as of 2018 [25-27]. According to the Savannakhet Health Provincial Office, Sepone and Vilabuly each have a district hospital, with 14 health centers in Sepone and 12 in Vilabuly [28].

Out of the 88 villages in Sepone, 19 intervention sites were selected in the catchment areas of five health facilities. These sites are called "model villages," as they previously implemented the 2017-2019 phase of the JICA Grassroot Project to promote ANC and facility-based delivery [29]. Since the project aims to extend the pairing of female and male VHVs/VHWs to all villages of Sepone District by 2024, we selected 18 control sites in the catchment areas of five health facilities of Vilabuly out of its 73 villages. The list of intervention and control villages in Sepone District and Vilabuly District is presented in Appendix B.

Participants

The target population of the quantitative component of the study is women aged 15-49 years who reside at the study sites. The participants will be women who gave birth within 12 months at the time of both the baseline and endline surveys. Women whose births resulted in stillbirths, those who did not consent to participate in the surveys, or those who faced a language barrier with surveyors - due to the presence of ethnic minorities who speak different local languages - will not be enrolled. In contrast, the qualitative component targets additional groups, such as village health volunteers and workers, male partners, family members, and health center staff.

Sample size and sampling method

We estimated the sample size using the logistic regression formula n > 10 m, where the minimum "n" of respondents with or without the study outcome is 10 times the number of covariates "m" to be included in the regression [30]. We estimated at least 80 facility-based follow-up PNC users with eight potential covariates. In 2022, VHVs/VHWs reported that there were 302 children of ≤1 year of age in the 37 targeted villages, including 137 from the 19 villages in Sepone and 165 from the 18 villages in Vilabuly. Due to the relatively low yearly delivery at the target sites, we will conduct exhaustive sampling at the baseline to include all eligible subjects, ensuring representative results [31].

For the end-line survey, we estimated the required sample size for difference-in-differences analyses to assess changes in the primary outcome before and after the intervention. We anticipate a 15.0% increase in facility-based follow-up PNC in the intervention villages and a 5.0% increase in the control villages over two years of implementation. Using a two-tailed z test for the difference between proportions, with 80% statistical power at the 5% significance level, unequal cluster size assessment, a design effect of 2, and a non-response rate of 10%, we estimated a sample size of 302 for the end-line survey [32,33].

Finally, at least 12 participants will be selected purposively for interviews in the qualitative component [34]. The enrollment of further participants will depend on when data saturation is reached.

Intervention, monitoring, and assessments

Between July and September 2024, a baseline survey will be conducted to assess pre-intervention levels of service utilization, women's socioeconomic and geographic characteristics, satisfaction with VHVs/VHWs, and other covariates related to the uptake of facility-based follow-up PNC visits. By November 2024, female-male VHV/VHW pairs were formed and trained on the intervention content at targeted sites in Sepone. Where necessary, new VHVs/VHWs will be selected following the national guidelines. The requirements include being a citizen of the Lao PDR, age ≥18 years, and being a resident of the village where they will serve [35]. In Vilabuly, we will ensure that each selected village has an active VHV/VHW, but no training will be conducted, as we aim to compare the intervention to the standard implementation of VHV/VHW activities.

Over the following 18 months, pregnant and postpartum women at all study sites will receive regular visits from VHVs/VHWs, either individually or in groups, for health counseling and educational activities focused on maternal health services, particularly postpartum care. In intervention sites, visits will occur at least once during the first and second trimesters of pregnancy, twice in the third trimester, and once during the first six weeks postpartum. VHVs and VHWs will highlight the benefits of facility-based follow-up PNC visits, extended postpartum stays at facilities, and the importance of husbands and family support during maternal care. While the main role of female VHVs/VHWs is to engage with mothers, male VHVs/VHWs in Sepone will be expected to have at least two interactions with male partners or family members during the third trimester of pregnancy and the first six postpartum weeks. The training content and frequency of visits by VHV/VHW will be validated using local recommendations to ensure policy alignment and greater community acceptance. In addition to skills related to MCH promotion, VHVs/VHWs in the intervention will receive training in task sharing and communication.

Around July 2026, an end-line survey will be conducted at all study sites to assess changes in outcome levels, and qualitative interviews will explore communities' perceptions and acceptance of paired VHVs/VHWs. 

To monitor implementation fidelity, VHVs/VHWs will complete standard reporting forms each month, documenting home visits and activities conducted. A midterm review using both monitoring data and qualitative insights will assess progress and inform any necessary course corrections.

Data collection

Both baseline and end-line assessments will be carried out using household surveys. Data will be collected by trained research assistants (medical students) using pretested semi-structured questionnaires prepared in English and translated into Lao using the Epi Info software. The questionnaires will be administered on tablets in Lao or other local languages as needed. The questions will focus on participants' socioeconomic and demographic characteristics, prior MCH service usage, and indicators of accessibility to healthcare facilities. Self-reported service utilization will be cross-validated using the maternal and child health handbooks (also known as the “pink book”).

Variables

WHO recommends that postpartum women have at least four postnatal care sessions regardless of delivery location, including within 24 hours after birth, on day 3, between seven days and 14, and after six weeks [11]. The primary outcome of the quantitative component is the use of at least one facility-based follow-up PNC visit within six weeks postpartum (yes/no). The secondary outcome is the duration (in hours) of facility-based postpartum stay by mothers after delivery. The two main predictor variables identified include residing in intervention versus control villages, and the frequency of VHVs/VHWs visits to mothers [36]. Additional variables will include respondents' sociodemographic characteristics, husbands' characteristics, household characteristics, gender norms, and trust in VHVs/VHWs.

Qualitative data will be gathered through key informant interviews and focus group discussions (FGDs). Interview and FGD guides will be developed in Lao, and interviewers (medical students) will receive training in qualitative data collection techniques. With participants’ consent, all interviews and discussions will be audio recorded, anonymized, and securely stored on a drive accessible only to the research team. The qualitative study will be conducted during the implementation phase to examine how the intervention is delivered and perceived, and to gain insights into the processes influencing its effectiveness. Interview and discussion guides will focus on the dynamics of interactions between VHVs/VHWs and mothers, between VHVs and mothers’ family members, and among VHVs who work together.

Data analysis

We will conduct difference-in-differences analyses using estimated levels of key indicators before and after the intervention, and test the significance of changes in outcomes. Pearson chi-square tests or t-tests will be used, as appropriate, to compare gaps in service use based on women's characteristics and other covariates. For multivariate analyses, mixed-effects logistic regression will be used to evaluate the associations between the primary outcome and predictor variables, while including interaction terms between time and group to assess the intervention effect. Statistics will be reported along with their 95% confidence intervals. P values of <0.05 will be considered to indicate statistical significance. All analyses will be performed using Stata Statistical Software (release 17, 2021, StataCorp, College Station, TX).

For the qualitative component, interview and FGD audio recordings will be transcribed, translated into English, and analyzed using both inductive and deductive content analysis approaches. Relevant codes will be extracted and organized into themes and sub-themes to provide insights into the intervention’s implementation context, feasibility, and acceptance within the community.

Ethical considerations

Ethical approval was obtained from the Ethics Committee of the University of the Ryukyus for Medical and Health Research Involving Human Subjects (approval no: 24-2329-00-00-00) and the Lao Tropical Medicine and Public Health Institute (approval no: 31/NECHR). The participants will be provided with comprehensive information sheets and consent forms outlining the purpose of the study. Participants aged 15-17 years will be requested to sign an assent form in addition to permission from their legal guardians. All the data collected will be handled anonymously, and confidentiality will be assured. Qualitative data, such as audio recordings, will be encrypted and stored securely on drives accessible only to the research team.

## Results

At the end of the study, several outcomes are anticipated in line with the study objectives. First, assuming baseline utilization is about 5% in both districts, we expect a 15% increase in Sepone (intervention) and a 5% increase in Vilabuly (control), resulting in a 10% difference in the proportion of women receiving at least one facility-based follow-up PNC visit. Second, guided by the theory of change, we hypothesize this improvement to result from enhanced counseling and more frequent interactions between mothers, male partners, and VHVs/VHWs through a gender-norms-sensitive approach. Specifically, regular household visits by paired VHVs/VHWs are expected to strengthen trust and satisfaction among mothers and promote male partner involvement, thereby contributing to increased PNC service uptake. Lastly, we also anticipate a longer average duration of postpartum facility stays among mothers in intervention areas.

## Discussion

In ethnic minority communities with strong patriarchal norms, women often face restrictions that limit their interactions with male CHWs, especially on sensitive topics like reproductive and maternal health [37-39]. In such settings, female CHWs are often preferred, as women are generally seen as mainly responsible for childcare [37]. Likewise, cultural norms may prevent CHWs, particularly females, from interacting with men outside their families, limiting communication with male household heads who play a key role in healthcare decisions [37]. Pairing male and female CHWs can help overcome these challenges by enabling more culturally appropriate communication. In Lao communities, this is important, as most VHVs/VHWs are men due to higher literacy rates, but are often less accepted when engaging directly with women on sensitive health issues. Male CHWs can focus on engaging male partners and handling literacy-based tasks like reporting, while female CHWs support mothers through counseling and education. This approach may strengthen both maternal care and male involvement in ways that respect local culture. 

Therefore, the present quasi-experimental study aims to estimate the effectiveness of female and male VHVs/VHWs working as pairs in increasing the uptake of facility-based follow-up PNC services among ethnic minorities in the Lao PDR.

Strengths and limitations

The study has several strengths. To our knowledge, it is the first in the Lao PDR to assess the effectiveness of a community-based intervention involving CHWs to improve continuity of care between health facilities and mothers, while addressing barriers related to gender norms. The pairing of male and female CHWs in this intervention aligns with the Lao National Primary Health Care Policy’s goal of rethinking strategies for delivering primary healthcare through VHVs/VHWs [15]. Moreover, implementing the intervention in collaboration with the Savannakhet Health Provincial Office increases the likelihood of local ownership, while the selection of sites ensures that the study recommendations are relevant to many other settings in Lao PDR, where VHVs/VHWs are predominantly male [8,9].

Despite its strengths, this study has a few potential limitations. First, the choice of a quasi-experimental design was made due to feasibility constraints; however, the lack of randomization increases the risk of selection bias, where systematic differences between groups may impact the results. Efforts were made to select target sites with sociodemographic and cultural similarities to minimize these differences, yet confounding factors such as varying healthcare access may still affect the outcomes. Second, as key factors will be assessed through self-reports, this may introduce reporting biases. Nonetheless, the validation of service utilization will be confirmed through mothers' medical handbooks.

## Conclusions

The Lao PDR has committed to expanding access to MCH services by promoting collaboration between male and female VHVs/VHWs. However, the extent to which this strategy can reduce gender norm-related barriers, particularly in ethnic minority communities, remains uncertain. This study aims to provide evidence to support the government’s approach by assessing the effectiveness of pairing male and female CHWs in improving key outcomes, including the uptake of facility-based follow-up postpartum care. In addition, it will document lessons learned from the intervention’s implementation to inform future scale-up.

## Figures and Tables

**Figure 1:**
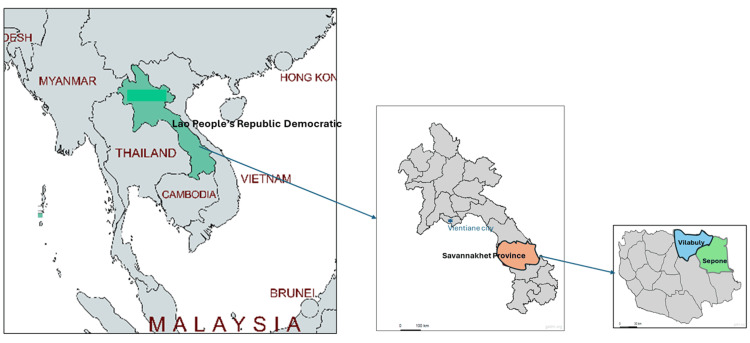
Map of study sites in Lao People's Democratic Republic (Lao PDR)

## Appendices

Appendix A

Appendix B 

**Table 1:** List of intervention and control villages

Intervention arm villages (Sepone District)	Control arm villages (Vilabuly District)
Huaycheangkao	Keangleknuea
Mueangchannok	Kokmark
Keangthongnok	Phaphilarng
Kaloukkao & Kaloukmai	That
Dongnoy	Nater
Kheaving	Nabalu
Vangyeangnoy	Poungpor
Keangpeayai	Numlap
Keangluangnok	Nummee
Arlainoy	Keanglektai
Sadueng	Thangbeangkeangluang
Kadub	Padong
Keangkiew	Huaydarng
Munchy	Torhuea
LaAorkao	Huaysuan
Panga	Lardeangnoy
Sawed	Dongyang
Vangmorthoum	Numkheep
Poung	
